# Somatic mosaic truncating mutations of *PPM1D* in blood can result from expansion of a mutant clone under selective pressure of chemotherapy

**DOI:** 10.1371/journal.pone.0217521

**Published:** 2019-06-26

**Authors:** Borahm Kim, Dongju Won, Seung-Tae Lee, Jong Rak Choi

**Affiliations:** Department of Laboratory Medicine, Yonsei University College of Medicine, Seoul, Korea; Odense University Hospital, DENMARK

## Abstract

**Background:**

*PPM1D* (Protein phosphatase magnesium-dependent 1δ) is known as a damage response regulator, a part of the p53 negative feedback loop. Truncating mutations of *PPM1D*, resulting in overexpression, are frequently found in the blood of patients with breast or ovarian cancer. To identify whether the *PPM1D* mutation predisposes patients to such cancers or if it results from the cancer and therapy, somatic *PPM1D* mutations in association with previous cancer and chemotherapy need to be explored.

**Methods:**

We performed next-generation sequencing (NGS) analysis of blood samples from patients suspected to have hereditary cancer. We grouped the patients according to their diagnoses and history of chemotherapy. For the patients with *PPM1D* mutations in blood, tumor tissue specimens were examined for the *PPM1D* mutation using conventional sequencing.

**Results:**

A total of 1,195 patients, including 719 patients with breast cancer and 240 with ovarian cancer, were tested, and four (~0.3%) had the truncating mutation in *PPM1D*. All truncating mutations were in exon 6, in mosaic form, with a mean allele fraction of 11.15%. While 395 out of the 1,195 patients had undergone chemotherapy, the four with the truncating mutation had a history of cisplatin-based chemotherapy. No corresponding mutations were identified in the tumor tissues.

**Conclusions:**

We investigated the frequency of the somatic mosaic *PPM1D* mutation, in patients with breast or ovarian cancer, which is suggested to be low and related to a history of cisplatin-based chemotherapy. It may be a marker of previous exposure to selective pressure for cells with an impaired DNA damage response.

## Introduction

Protein phosphatase magnesium-dependent 1δ (*PPM1D*) encodes the Wip1 phosphatase and is known as a regulator of the DNA damage response pathway. Wip1 dephosphorylates the tumor suppressor p53, CHK1, CHK2 and other key components of the pathway as part of a negative feedback regulatory loop [1,2]. Induced by p53 initiating cell cycle arrest or apoptosis upon cellular stress, Wip1 downregulates p53 level and drives the cell back to its normal state [2].

Gain-of-function mutations of *PPM1D* have been reported in a wide variety of cancers, including breast cancer and ovarian cancer [3–10]. Over suppression of p53 releases the cell from arrest to proceed the cell cycle, resulting in carcinogenesis [11]. Although copy number gain and increased expression level of *PPM1D* have been reported, [4,7,10], it has also been reported that truncating mutations in exon 6 of *PPM1D* lead to its overexpression, escaping from the control of degradation signaling [3,11]. In recent studies, these truncating mutations of *PPM1D* in mosaic form were found in the blood with higher frequency among patients with ovarian cancer or breast cancer than in the control group [5,12], suggesting a causal relationship between the mutations and the cancers. In contrast, it has also been reported that *PPM1D* mosaic truncating mutations in the blood have been associated with chemotherapy rather than predisposition to the cancers [9,13].

To support this idea, the association of somatic *PPM1D* mutations with previous cancer and chemotherapy needs to be explored. In this study, we investigated the prevalence and characteristics of the truncating *PPM1D* mutation among patients with cancer by using NGS hereditary cancer panel testing. We analyzed the somatic truncating *PPM1D* mutation status in the context of previous chemotherapy.

## Methods

### Patients and samples

A total of 1,195 peripheral blood samples of patients suspected to have had hereditary cancer according to National Comprehensive Cancer Network guideline (https://www.nccn.org/professionals/physician_gls/pdf/genetics_screening.pdf, registration required) in Severance Hospital between July 2016 and July 2018 were subjected to NGS gene panel testing for hereditary cancer. For those patients, investigation of truncating mutations of *PPM1D* was performed. For the three cases with identified *PPM1D* truncating mutations, corresponding formalin-fixed paraffin-embedded primary cancer specimens were subjected to mutational analysis. Written form of informed consent was obtained for all patients. The current study was approved by Severance Hospital Institutional Review Board (4-2019-0021).

### DNA extraction and sequencing

Genomic DNA was extracted from peripheral blood using the QIAamp DNA Blood Mini Kit (Qiagen, Venlo, The Netherlands). Approximately 500 ng of genomic DNA was fragmented into segments between 150 and 250 bp long, using the Bioruptor Pico Sonication System (Diagenode, Liège, Belgium), which were then end repaired and ligated to Illumina adapters (Illumina, San Diego, CA, USA) and indices. Sequencing libraries were then hybridized with the capture probes (Celemics, Seoul, Korea). All procedures were performed per the manufacturer’s instructions. Enriched DNA was then amplified, and clusters were generated and sequenced on a NextSeq 550 instrument (Illumina) with 2×151 bp reads. NGS tests were performed using a custom gene panel consisting of 65 genes related to hereditary cancer (S1 Table). For genomic DNA extraction of tumor samples, Maxwell RSC DNA FFPE Kits (Promega, Madison, WI, USA) were used and Sanger sequencing of the specimen was performed.

### Data analysis and interpretation

Reads were aligned using the Burrows-Wheeler Alignment tool (0.7.12) on human genomic reference sequences (GRCh37) [14]. To identify single nucleotide sequence variations, the HaplotypeCaller in the Genome Analysis Toolkit package (3.8–0) was used [15]. All mutations were annotated using ANNOVAR and VEP (87) software [16,17]. Detected variants were further examined by visual verification using the Integrative Genomics Viewer [18]. The pathogenicity of variants was classified according to the American College of Medical Genetics and Genomics (ACMG) criteria [19].

## Results

### Sequencing data and patients

A total of 1,195 patients were enrolled. Among them, 725 patients had breast cancer (60.7%) and 246 had ovarian cancer (20.6%), as summarized in Table 1. Three hundred and ninety five patients out of 1,195 (33.1%) had received chemotherapy before genetic testing. The median depth of coverage of the gene panel was 691× with a maximum depth of 7,976×. The median depth of the *PPM1D* gene was 990x, ranging from 650x to 2,277x.

**Table 1:** Cancer types of people who have undergone NGS gene panel testing for hereditary cancer.

Type of cancer	n (%)
Breast	719 (60.2)
Ovarian	240 (20.1)
Breast and ovarian	6 (0.5)
Colorectal	134 (11.2)
Endometrial	15 (1.3)
Retinoblastoma	13 (1.1)
Pancreatic	12 (1.0)
Gastric	11 (0.9)
other	45 (3.8)
total	1195

Breast and/or ovarian cancer were the most common (80.6%)

### Truncating *PPM1D* mutations

Truncating mutations in *PPM1D* were detected in four patients (~0.3% of total cases), one (1/725, 0.14%) with breast cancer and three (3/246, 1.22%) with ovarian cancer (Table 2). All truncating mutations were in mosaic form on the cluster region within a 370-base-pair region in exon 6, the final exon of the gene [5]. The mean variant allele fraction (VAF) was 11.15%, ranging from 5.4% to 15.4%. Meanwhile, 15 missense variants of *PPM1D* were identified in each fifteen patient except the four harboring the truncating mutations in *PPM1D*. They were located in exon 1, 2, 5, and 6 and variant allele frequencies were approximately 0.5 (S2 Table). They were all variants with uncertain clinical significance according to the ACMG guideline.

**Table 2:** Characteristics of *PPM1D* truncating mutation carriers.

ID	Sex	Diagnosis	Age at diagnosis, y	Family history	DNA change	Affected protein	VAF	Median depth	Concurrent mutation (allele fraction)	*PPM1D* mutation in tissue sample	Chemotherapy regimen	The period since the use of chemotherapy
P1	M	Breast cancer, left / Lung cancer	82/66	Sibling, colon cancer	c.1423G>T	p.Glu475Ter	0.154	1380	None	Not detected	Cisplatin and etoposide	20 years
P2	F	Ovarian cancer	59	none	c.1434C>A	p.Cys478Ter	0.167	723	*BRCA1* (0.48)	Not detected	Carboplatin and paclitaxel	8 months
P3	F	Ovarian cancer	52	none	c.1619delA	p.Glu540GlyfsTer7	0.054	903	None	Not tested	Carboplatin, doxorubicin, cisplatin and belotecan	32 months
P4	F	Ovarian cancer	61	none	c.1396_1397delATinsTA	p.Ile466Ter	0.071	858	None	Not detected	carboplatin and paclitaxel	10 months

VAF, Variant allele frequency

The mean age at diagnosis of the *PPM1D* truncating mutation carriers was 63.5 years (range, 52–82 years), and they did not have family history of breast or ovarian cancer. One case (P2) had a concurrent truncating mutation in *BRCA1* (c.2345T>A, p.Leu785Ter), which is classified as a pathogenic variant according to the ACMG guideline, in addition to the truncating *PPM1D* mutation. The cancer type was invasive ductal carcinoma in a case with breast cancer and serous carcinoma in all cases with ovarian cancer (Table 2).

All four patients with a *PPM1D* mutation had a history of chemotherapy (odds ratio, 18.4; 95% confidence interval, 0.990–342.67; p = 0.051). Chemotherapy regimens included cisplatin or carboplatin for all four patients. The time interval between chemotherapy and the NGS test ranged from 10 months to 20 years.

### *PPM1D* mutation status in cancer specimen

We obtained the peripheral blood and tumor tissues of three mutation-positive cases (P1, P2 and P4) except for P3 whose specimen was not available. and performed conventional PCR and sequencing. All the low level *PPM1D* mutations in blood samples were identified by conventional sequencing, but no corresponding mutations in the tumor tissues were observed, as suggested in the previous reports [5,12] (S1 Fig).

## Discussion

*PPM1D* is known as a DNA damage response regulator, which is a part of the p53 regulatory feedback loop. *PPM1D* is induced by activated p53, and it dephosphorylates and inactivates p53 as well as other target proteins involved in DNA repair [20,21]. It negatively regulates the p53-mediated apoptosis and returns the cell to its original state [22].

Increased expression of *PPM1D* and p53 over suppression have been reported in various cancer tissues [4,7,10]. Notably, *PPM1D* truncating mutations are invariably found in exon 6 and are known to enhance its function [3,11]. This results in mRNA without the last exon, which is responsible for the degradation signal, making the molecule more stable. This leads to a gain of function effect by more strongly dephosphorylating its targets compared to the wild type dose [11,23]. Because PPM1D also dephosphorylates and inactivates other proteins working in the DNA damage repair system, the truncating mutations of *PPM1D* are assumed to have a role in carcinogenesis in various types of cancers.

Somatic mosaic mutations of *PPM1D* in the blood have been more frequently found in patients with various cancers including breast and ovarian cancers compared to control groups, raising concern that it has a possible role in cancer predisposition [5,9,12]. It is assumed that the cancer driver mutation is enriched in tumor tissue compared to other tissues such as blood; however, no corresponding mutations have been identified in tumor tissues in most cases [5,12].

In 2016, Pharoah et al. and Swisher et al. suggested that somatic mosaic mutations in *PPM1D* in the blood resulted from previous chemotherapy [13,24]. Platinum-based chemotherapy such as cisplatin is the basis of the chemotherapy regimen for ovarian and breast cancers. Cisplatin, in particular, is known to cross-link with DNA and cause DNA sequence changes at a high frequency [25]. The fact that most of the somatic mosaic mutations in *PPM1D* were observed in the blood of patients with ovarian and breast cancer supports the association of these mutations with platinum-based chemotherapy [13,24,26].

Our NGS hereditary cancer panel analysis with blood revealed somatic mosaic mutations of *PPM1D* in four out of 965 patients with breast and/or ovarian cancer (0.41%). The frequency was similar to that reported previously (0.32% in breast and/or ovarian cancer), and the mutations were also within the reported clustering region of exon 6, with no particular hotspots [5]. However, the percentage of individuals harboring the mutation among patients with lymphoid malignancy and prior chemotherapy was reported as high as 20% using more sensitive method, error-corrected sequencing [27]. Along with previous reports, the mutations present in low fractions in blood were not enriched or identified in the tumor tissues examined. Moreover, a patient had a concurrent pathogenic *BRCA1* mutation, which predisposes more strongly to the condition. These results could be supportive of mosaic *PPM1D* mutations resulting from treatment rather than causing a predisposition to breast or ovarian cancer. There were 395 patients who underwent previous chemotherapy, and all four patients with the *PPM1D* mutation received cisplatin-based chemotherapy (p = 0.051). Those four patients had undergone chemotherapy from 10 months to 20 years ago. Concluding that the *PPM1D* mutation predisposed patients to breast or ovarian cancer requires more evidence. In this context, somatic *PPM1D* mutations could be a result of expansion of a *PPM1D*-mutated clone under selective pressure by cisplatin chemotherapy, rather than a risk factor or cause of the respective cancers.

Indeed, somatic mosaic mutations of *PPM1D* have been reported in populations with clonal hematopoiesis of indeterminate potential (CHIP) [28,29]. In our study, the median age of the patients with *PPM1D* truncating variants was 60 (range, 52–82), which was a little higher than the age of patients with familial breast and ovarian cancer. Rather, clonal mosaic hematopoiesis could be common in this age group. *PPM1D* mutations are far from being common in the general population and their frequency is increasing in association with prior chemotherapy [28–30]. It was the most common mutation associated with CHIP in patients with cisplatin-based myeloablative therapy or therapy-related myeloid neoplasm (TMN) [26,30]. This is because the pre-existing or therapy-induced *PPM1D* mutated clones have survival advantages in the context of cisplatin therapy, resisting apoptosis and thus chemotherapy [26,31]. Considering the recent studies stating the association between the clonal *PPM1D* mutations in blood and TMN, the mutations could be associated with an increased risk for certain subtypes of hematologic malignancy [26,30]. According to the recent study, patients with CHIP showed a higher incidence of TMN and shorter overall survival among patients with myeloablative therapy [30]. Among them, patients bearing *PPM1D* as CHIP showed significantly poorer outcomes [30]. For better precision in treatment, the increased risk of such hematologic malignancy should be taken into account in long-term follow up.

## Conclusions

In conclusion, we investigated somatic mosaic *PPM1D* mutations in patients with various cancers, including breast and ovarian cancers and found that all four patients bearing the truncating mutations had a history of cisplatin-based chemotherapy. This suggests that these mutations may be due to the increase of a mutant clone under selective pressure by cytotoxic therapy.

## Supporting information

S1 Fig Confirmation of truncating mutations in blood and cancer specimens by Sanger sequencing. Samples from three (P1, P2 and P4) who had a low percentage of truncating mutations in peripheral blood by NGS were confirmed by Sanger sequencing. However, no corresponding mutations in primary tumor tissues were observed.
(TIF)

S1 Table Genes included in the custom NGS gene panel. (DOCX)

S2 Table Characteristics of *PPM1D* missense mutation carriers. (DOCX)
