# Supplementary figures and images for: Somatic mosaic truncating mutations of PPM1D in blood can result from expansion of a mutant clone under selective pressure of chemotherapy
Source: PLoS One. 2019 Jun 26;14(6):e0217521. doi: 10.1371/journal.pone.0217521 (PMC6594580; doi:10.1371/journal.pone.0217521)

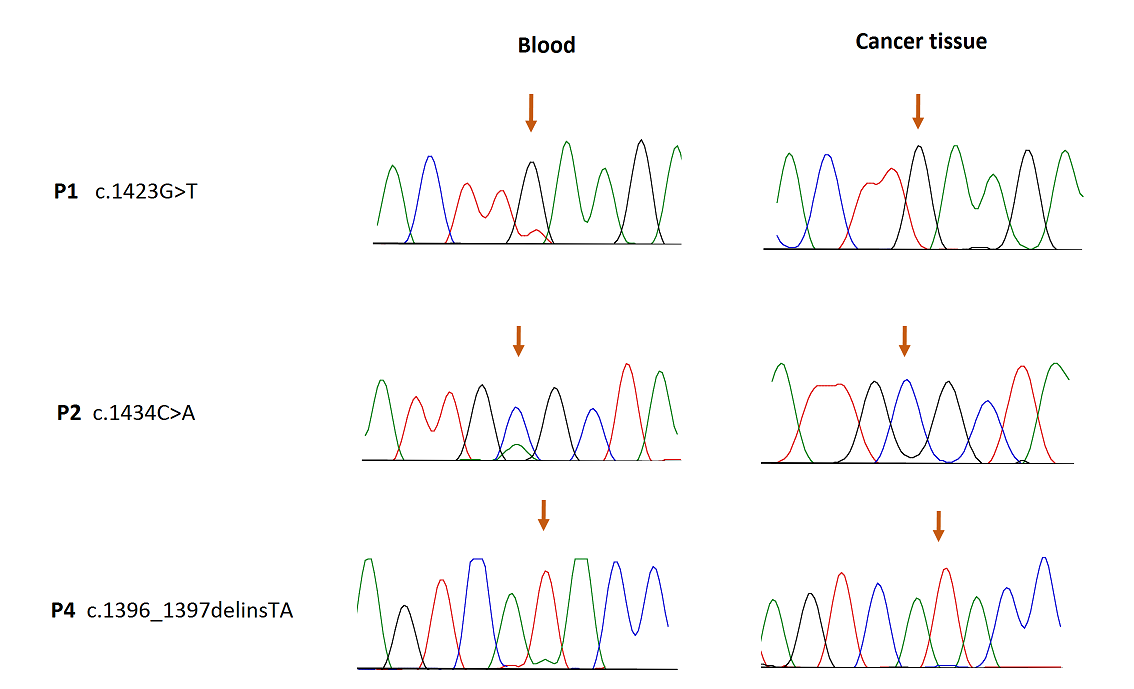

Supplement: S1 Fig — Samples from three (P1, P2 and P4) who had a low percentage of truncating mutations in peripheral blood by NGS were confirmed by Sanger sequencing. However, no corresponding mutations in primary tumor tissues were observed. (TIF) [file pone.0217521.s001.tif]
