# Supplementary material for: Somatic mosaic truncating mutations of PPM1D in blood can result from expansion of a mutant clone under selective pressure of chemotherapy
Source: PLoS One. 2019 Jun 26;14(6):e0217521. doi: 10.1371/journal.pone.0217521 (PMC6594580; doi:10.1371/journal.pone.0217521)
Supplement: S1 Table — (DOCX) [file pone.0217521.s002.docx]

**Supplementary Table 1.** Genes included in the custom NGS gene panel

*ALK, APC, ATM, AXIN1, AXIN2, BARD1, BLM, BMPR1A, BRCA1, BRCA2, BRIP1, CDH1, CDK4, CDKN2A, CHEK2, CTNNB1, EPCAM, EXO1, FANCM, FLCN, GALNT12, GPC3, GREM1, KIF1B, KRAS, LMO1, MEN1, MLH1, MLH3, MRE11A, MSH2, MSH6, MUTYH, NBN, NF1, NF2, NRAS, NTRK1, PALB2, PAX6, PHOX2B, PMS1, PMS2, POLD1, POLE, PPM1D, PRSS1, PTCH1, PTEN, RAD50, RAD51, RAD51C, RAD51D, RB1, RET, RUNX1, SDHA, SDHAF2, SDHB, SLX4, SMAD4, STK11, TP53, VHL, WT1*
