# Supplementary material for: Somatic mosaic truncating mutations of PPM1D in blood can result from expansion of a mutant clone under selective pressure of chemotherapy
Source: PLoS One. 2019 Jun 26;14(6):e0217521. doi: 10.1371/journal.pone.0217521 (PMC6594580; doi:10.1371/journal.pone.0217521)
Supplement: S2 Table — (DOCX) [file pone.0217521.s003.docx]

**Supplementary Table 2. Characteristics of *PPM1D* missense mutation carriers.**

| Sex | Diagnosis | Age at diagnosis, y | Family history | Exon | DNA change | Affected protein | VAF | Median depth | Concurrent mutation | Chemotherapy regimen | The period since the use of chemotherapy |
| --- | --- | --- | --- | --- | --- | --- | --- | --- | --- | --- | --- |
| F | Breast cancer, right | 52 | none | 6 | c.1787A>G | p.His596Arg | 0.46 | 1288 | None | None | N.A |
| F | Breast cancer, left | 44 | father, leukemia | 6 | c.1550C>T | p.Thr517Ile | 0.50 | 1358 | None | None | N.A |
| F | Breast cancer, left | 62 | none | 6 | c.1550C>T | p.Thr517Ile | 0.50 | 1266 | None | None | N.A |
| F | Retinoblastoma | 1 | none | 6 | c.1328A>G | p.Asn443Ser | 0.57 | 450 | None | carboplatin, vincristin, and etoposide | 1 month |
| F | Endometrial cancer | 60 | none | 6 | c.1787A>G | p.His596Arg | 0.44 | 997 | None | None | N.A |
| F | Breast cancer, left | 35 | mother, thyroid cancer | 6 | c.1343A>G | p.Asn448Ser | 0.50 | 675 | None | None | N.A |
| F | Breast cancer, right | 43 | sibling, ovarian cancer  mother, peritoneal cancer | 6 | c.1407A>C | p.Lys469Asn | 0.48 | 1706 | None | cyclophosphamide and doxorubicin | 2 years |
| F | Breast cancer, right | 40 | sibling, breast cancer | 6 | c.1706C>A | p.Thr569Asn | 0.46 | 1411 | None | None | N.A |
| F | Breast cancer, both | 47 | mother, lung cancer | 6 | c.1594A>G | p.Thr532Ala | 0.46 | 1455 | None | None | N.A |
| F | Breast cancer, left | 50 | father, melanoma | 5 | c.1037G>T | p.Cys346Phe | 0.48 | 504 | None | None | N.A |
| F | Breast cancer, both | 45 | father, stomach cancer | 5 | c.1037G>T | p.Cys346Phe | 0.47 | 195 | None | None | N.A |
| F | Breast cancer, left | 65 | sibling, breast cancer | 5 | c.1037G>T | p.Cys346Phe | 0.47 | 250 | None | None | N.A |
| F | Breast cancer, right | 45 | sibling, breast cancer | 2 | c.473C>A | p.Ala158Glu | 0.54 | 514 | None | None | N.A |
| F | Breast cancer, right | 58 | sibling, breast cancer | 1 | c.117C>T | p.Pro39= | 0.50 | 1120 | None | None | N.A |
| F | Breast cancer, left | 32 | none | 1 | c.17C>T | p.Ser6Leu | 0.48 | 1922 | None | None | N.A |

VAF, Variant allele frequency; N.A, Not applicable
